# *In situ* X-ray crystallographic study of the structural evolution of colloidal crystals upon heating

**DOI:** 10.1107/S0021889813003725

**Published:** 2013-06-07

**Authors:** A. V. Zozulya, J.-M. Meijer, A. Shabalin, A. Ricci, F. Westermeier, R. P. Kurta, U. Lorenz, A. Singer, O. Yefanov, A. V. Petukhov, M. Sprung, I. A. Vartanyants

**Affiliations:** a Deutsches Electronen-Synchrotron (DESY), Notkestrasse 85, D-22607 Hamburg, Germany; b Van ’t Hoff Laboratory for Physical and Colloid Chemistry, Debye Institute, University of Utrecht, Padualaan 8, 3508 TB Utrecht, The Netherlands; c Center for Free-Electron Laser Science, DESY, Notkestrasse 85, D-22607 Hamburg, Germany; d National Research Nuclear University ‘MEPhI’, 115409 Moscow, Russian Federation

**Keywords:** colloidal crystals, small-angle X-ray scattering, thermal treatment

## Abstract

The structural evolution of colloidal crystals made of polystyrene hard spheres has been studied *in situ* upon incremental heating of a crystal in a temperature range below and above the glass transition temperature of polystyrene. Thin films of colloidal crystals having different particle sizes were studied in transmission geometry using a high-resolution small-angle X-ray scattering setup at the P10 Coherence Beamline of the PETRA III synchrotron facility. The transformation of colloidal crystals to a melted state has been observed in a narrow temperature interval of less than 10 K.

## Abstract 2

The results of a real-time X-ray crystallographic study of the melting transition of polystyrene colloidal crystals during heating are presented.

## 1. Introduction

Colloidal crystals are close-packed self-assembled arrangements of monodisperse colloidal particles of sub-micrometre size. These systems, typically composed of either silica or polymer colloids, can diffract light in visible and near-infrared regions depending on the particle size. Structures templated by these crystals can possess a photonic bandgap preventing light from being transmitted in a certain range of frequencies (Vlasov *et al.*, 2001 ▶). In contrast to costly lithographic schemes of nanofabrication of photonic crystals, colloidal crystals can be synthesized under ambient conditions by vertical deposition techniques (Hilhorst *et al.*, 2009 ▶; Meijer *et al.*, 2012 ▶) and thus represent a cheap alternative class of materials with potential applications in photonics and optoelectronics [see special issue on electromagnetic crystal structures, design, synthesis and applications edited by Scherer *et al.* (1999) ▶ in *J. Lightwave Technol.*].

It has been shown that, by sintering and annealing a three-dimensional colloidal crystal at elevated temperatures, it is possible to modify the photonic bandgap properties (Míguez *et al.*, 1998 ▶; Gates *et al.*, 2000 ▶; Deng *et al.*, 2011 ▶). Furthermore, the glass transition temperature for polymers is known to be greatly influenced by free surfaces (Keddie *et al.*, 1994 ▶; Forrest *et al.*, 1996 ▶; Bäumchen *et al.*, 2012 ▶). After transferring a free-standing polystyrene film to a substrate, and thus reducing the free-surface-to-volume ratio by a factor of two, an increase of 50 K of the glass transition temperature has been observed (Bäumchen *et al.*, 2012 ▶). In a range of temperatures below the glass transition temperature of a polymer, the crystal retains a long-range order and undergoes a blue shift of the optical attenuation bands. The temperature region beyond the long-range-ordered phase, when the crystal starts to deteriorate, is not well studied, although it has important technological aspects regarding the tolerable temperature range of a photonic device. The physical processes taking place during heating and annealing of the colloidal crystal involve particle shape change, thermal shrinkage, interpolymer diffusion and particle melting (Míguez *et al.*, 1998 ▶; Gates *et al.*, 2000 ▶; Hu *et al.*, 2010 ▶).

Several methods can be used to monitor the structure of colloidal systems. In the case of large colloidal particles (larger than 0.5 µm) it is possible to image directly the structure and defects in colloidal crystals using optical microscopy methods (Dinsmore *et al.*, 2001 ▶; Schall *et al.*, 2004 ▶). For particle sizes smaller than 500 nm the resolution of optical microscopy comes to a limit and alternative methods have to be used. Transmission and scanning electron microscopy provide unprecedented spatial resolution. However, electron microscopy studies typically involve elaborate sample preparation and can only access the surface structure of a colloidal crystal because of the low penetration depth of electrons. In contrast, high-resolution X-ray scattering and diffraction provide a well established way for nondestructive characterization of crystalline and amorphous materials (Pietsch *et al.*, 2004 ▶). Owing to their high penetration depth and nondestructive characterization approach, X-ray scattering techniques offer favourable conditions for *in situ* studies.

When applied to colloidal crystals, the complete crystallographic description of the structure and defects of thin colloidal films is possible using high-resolution small-angle X-ray scattering based on the use of highly intense and collimated synchrotron X-rays (Dolbnya *et al.*, 2005 ▶; Petukhov *et al.*, 2006 ▶; Hilhorst *et al.*, 2009 ▶; Byelov *et al.*, 2010 ▶). Recent studies using coherent X-ray diffraction have shown possibilities for *ab initio* reconstruction of the two-dimensional and three-dimensional arrangement of colloidal spheres in a crystal (Gulden *et al.*, 2010 ▶, 2012 ▶; Zozulya *et al.*, 2012 ▶). In this work, we present *in situ* crystallographic studies of melting transitions in polystyrene colloidal crystals with different particle sizes upon incremental heating in a range of temperatures below and above the glass transition temperature of bulk polystyrene (373 K).

## 2. Experimental

**Figure 1:**
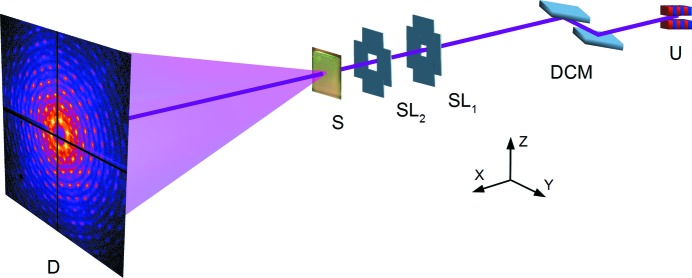
Schematic layout of the experiment. A white beam from an undulator source (U) is monochromated by a double-crystal monochromator and shaped using a pair of slits (SL_1_, SL_2_). The sample (S) is located at 87.7 m distance from the source. Diffraction patterns are recorded by a two-dimensional detector (D) positioned at 5.2 m distance behind the sample.

The experiments have been performed at the P10 Coherence Beamline of the PETRA III synchrotron facility at DESY, Hamburg, Germany. The P10 beamline is dedicated to coherent X-ray scattering experiments utilizing photon correlation spectroscopy and coherent diffraction imaging techniques in the photon energy range between 4 and 20 keV (the P10 beamline web page; http://hasylab.desy.de/facilities/petra_iii/beamlines/p10_coherence_applications/index_eng.html). The schematic layout of the experimental setup is shown in Fig. 1 ▶.

A white beam from an undulator (U) was monochromated using a vertical double-crystal Si(111) monochromator. The photon energy of 15 keV was selected for the experiment in order to assure high penetration depth through the colloidal crystal sample and cover a sufficient number of higher diffraction orders by a two-dimensional detector. The undulator source has a full width at half-maximum size of 14 × 85 µm (vertical × horizontal) and beam divergence of 4 × 28 µrad. The sample (S) was positioned at 87.7 m distance from the source and a pair of slits separated by 0.5 m along an optical axis was used to define the beam footprint on a sample. The beam size was defined by the first slit (SL_1_) located 0.8 m upstream, while the second slit (SL_2_) was used as a guarding aperture to reduce background scattering of an incident X-ray beam. In our experiment, the beam-defining slit was set to a size of 50 × 50 µm and the guard slit was set to 180 × 180 µm which provided an incident photon flux of 1.7 ×10^10^ photons s^−1^ on a sample.

The colloidal crystals were synthesized by vertical deposition of colloidal particles of linear polystyrene on thin glass slides. Colloidal suspensions contained particles at a volume fraction of 1.0% in water. Vertical deposition was performed on thin glass slides at 323 K for at least 24 h. The resulting colloidal crystal films consisted of 40–50 monolayers depending on the position along a sample and the particle size. The glass substrates with crystals were cut into 3 × 6 mm pieces and mechanically attached to a copper holder plate. The copper plate was fixed to a copper block designed as an insert flange in the vacuum sample chamber. The heating of the copper block was supplied by two parallel connected heating elements built into the copper block body. In order to provide sufficient heat exchange, the inner copper block with heating elements was separated by a Peltier element from the outer copper part connected to a water cooling cycle. The temperature of the sample holder was measured by two PT100 temperature sensors integrated into the copper block. Temperature and heating power controls were performed using a LakeShore 340 temperature controller.

**Figure 2:**
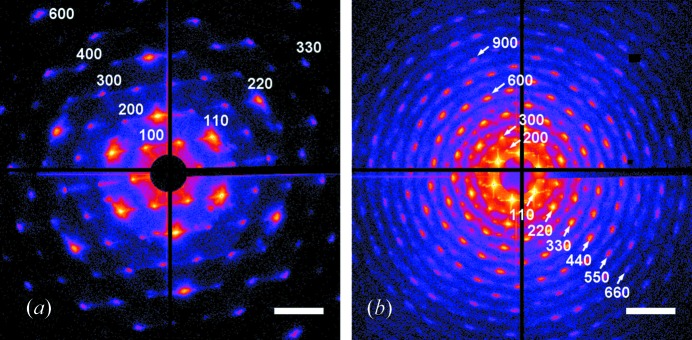
Diffraction patterns from colloidal crystals with particle sizes of (*a*) 163 nm (sample A) and (*b*) 430 nm (sample B) measured at room temperature. Reflections of 100 and 110 type are indexed on these plots. Hereafter the intensity colour scale is logarithmic and the scale bar is 6 × 10^−3^ Å^−1^.

The sample was aligned perpendicular to the beam in transmission geometry and X-ray diffraction patterns were acquired using the pixel array detector MAXIPIX. The MAXIPIX detector module consists of four chips (2 × 2 configuration) with a pixel size of 55 µm and has a total area of 516 × 516 pixels (Ponchut *et al.*, 2011 ▶). The gap of six pixels between adjacent chips results in a thin cross-like region of missing data in the measured diffraction patterns (Figs. 2 ▶ and 3 ▶). The detector was placed in a far-field position at 5.2 m distance downstream of the sample, thus providing a resolution of 8 × 10^−5^ Å^−1^ in reciprocal space. To eliminate the air scattering background, an evacuated flight tube covered the beam path between sample and detector. The detector was protected from the directly transmitted beam by a circular beamstop of 3 mm diameter mounted on a wire and positioned inside the flight tube.

## 3. Results

In order to investigate the dependence of the particle size on the structural evolution upon heating, two samples with polystyrene particle sizes of 163 nm (sample A) and 430 nm (sample B) were chosen for the experiment. Before starting the thermal treatment, the specimen was raster scanned transversely to the beam over an area of 1 mm^2^ with a step of 100 µm. The optimal position on a crystal was selected by evaluating the diffraction patterns in terms of sample homogeneity, number of resolved diffraction maxima and their intensity. In the experiment, the temperature of a sample was increased in steps starting from room temperature (293 K). In the range from 293 to 393 K we used temperature increments of 5 K, and the further temperature increase was performed with an increment of 1 K. For temperature stabilization a waiting time of 5 min was applied at each temperature point before starting the data collection. The diffraction patterns were collected at each temperature by acquiring a series of 500 frames with an exposure time of 0.1 s per frame. The resulting diffraction patterns were obtained by summing up all frames in a series and normalizing the result to the total number of frames.

In order to evaluate the crystal quality of intact samples, the diffraction patterns from both samples were measured at room temperature (Fig. 2 ▶). Indexing of Bragg reflections was performed using a hexagonal set of basis vectors as reported by Dolbnya *et al.* (2005 ▶). The observed sixfold symmetry of 100- and 110-type Bragg reflections clearly indicates a hexagonal close-packed structure present in both colloidal crystal samples. For the smaller spheres of sample A a set of 100 reflections up to the sixth order and a set of 110 reflections up to the third order can be observed in Fig. 2 ▶(*a*). For the larger spheres of sample B a set of 100 reflections up to the 12th order and a set of 110 reflections up to the sixth order were observed (Fig. 2 ▶ *b*). Intensity modulations up to the 15th order are also clearly observed owing to the form factor of a 430 nm spherical particle. Despite the two times difference in reciprocal space areas covered by the detector for the two samples, the observed ratios of diffraction peak intensities indicate a higher scattering power of larger particles and some difference in crystal quality in favour of sample B. Indeed, the two crystals were fabricated from colloidal suspensions of different polydispersity, 2.7% for the 163 nm particles and 2.1% for the 430 nm spheres. It is interesting to note that the intensity distribution around strong 110 reflections is partially modulated by the direct beam shape. Namely, the crossed streaks produced by the beam-defining slits can be seen around the 110 and 220 reflections for the 430 nm colloidal crystal (Fig. 2 ▶ *b*).

**Figure 3:**
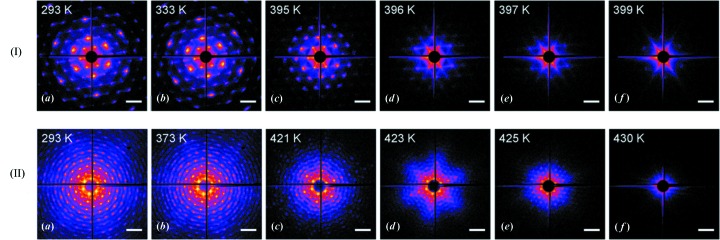
*In situ* diffraction patterns from sample A (top row) and sample B (bottom row) measured during incremental heating of the colloidal crystal. The sample temperature is marked at the top left corner for each of the displayed patterns.

The diffraction patterns measured from the colloidal crystals A and B at different temperatures in the process of incremental heating are shown in Fig. 3 ▶. The two samples show similar scenarios of structural evolution upon heating. At the beginning of the temperature increase the diffraction patterns do not reveal any substantial structural rearrangements, except for small azimuthal shifts of the whole diffraction pattern. Apparently, these changes indicate that crystalline domains constituting a colloidal film undergo slight relaxation, observed as an azimuthal tilt in an angular range of several degrees, which is driven by the first stages of crystal heating. Upon reaching a certain temperature (∼363 K) the diffraction patterns were stabilized and no changes were observed over a wide temperature range. Further temperature increase leads to the deterioration and disappearance of higher diffraction orders. This is accompanied by the enhancement of the diffuse scattering distribution with hexagonal symmetry of apexes pointing along the 〈110〉 crystallographic directions [Figs. 3 ▶(*c*) and 3(*d*), in rows (I) and (II)]. Before the crystal starts to decompose, the whole colloidal crystal lattice undergoes a thermal shrinkage of 3–5% as deduced from the analysis of the 220 diffraction peak shift. Finally, the last diffraction peaks and diffuse scattering also decay with further temperature increase and disappear almost completely at temperatures of 400 and 430 K for samples A and B, respectively.

Interestingly, a sixfold azimuthal modulation of the diffuse scattering pattern develops upon temperature rise. This modulation, which is most pronounced for the 430 nm particles [Fig. 3 ▶(*d*), in row (II)] and present to a lesser extent for the 163 nm particles [Fig. 3 ▶(*d*), in row (I)], clearly points to the variation of the particle form factor, which is maximal along the 〈110〉 crystallographic directions. This indicates that particles in the lattice lose their spherical shape first and obtain a dodecahedron shape at 396 and 423 K for samples A and B, respectively. This change from a sphere to a dodecahedron has also been observed for polymethyl methacrylate particles when exposed to heat (Vutukuri, 2012 ▶). It is noteworthy that, even after losing the dodecahedron shape, the close-packed order is still maintained up to 397 K for sample A [Fig. 3 ▶(*e*), in row (I)] and up to 425 K [Fig. 3 ▶(*e*), in row (II)] for sample B. Apparently, most of the particle mass is still located on a lattice position even after the particle loses its shape.

**Figure 4:**
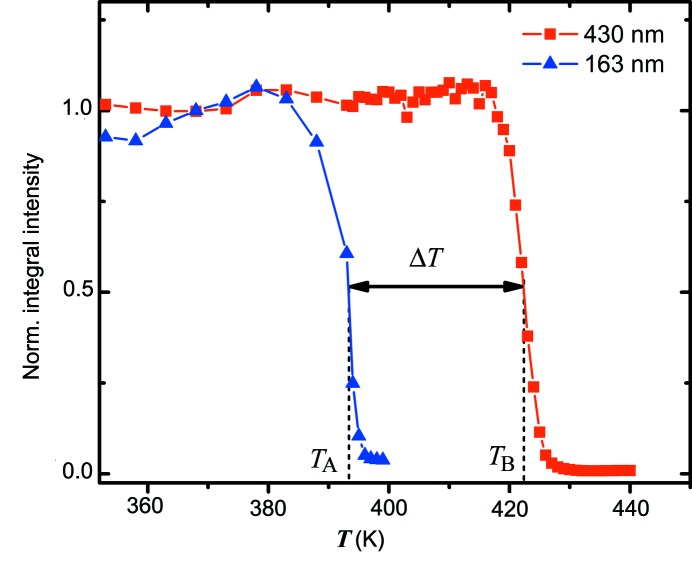
The dependence of total scattered intensity over a diffraction pattern as a function of sample temperature. Two specific temperatures, *T*_A_ and *T*_B_, corresponding to the melting transition for colloidal crystals of different particle sizes, are observed.

The experimental dependencies of total scattered intensity over a diffraction pattern as a function of sample temperature obtained for both investigated colloidal crystals are shown in Fig. 4 ▶. A sharp decay of integral intensity indicates the transition from crystalline to amorphous phase. The transition occurs in a narrow temperature interval of less than 10 K at temperatures *T*_A_ = 393 K for sample A and *T*_B_ = 422 K for sample B; thereby a transition temperature difference Δ*T* of about 30 K was observed. This observation is in agreement with the results of pioneering work by Keddie *et al.* (1994 ▶) on the dependence of the glass transition temperature on the thickness of a polymer film. In our study, sample B consisted of particles that are larger than those of sample A by a factor of 2.6, which results in the same proportion for the crystal thicknesses and the free-surface-to-volume ratios. Furthermore, we found out that sample B was of higher crystalline quality than sample A. This explains the observed difference in melting transition temperatures. It has to be mentioned that we studied the colloidal films deposited on glass substrates, which may result in an offset between the absolute temperatures of the film and the copper block due to the low thermal conductivity of the glass substrate. Therefore we restrict our study mainly to the relative temperature differences.

## 4. Conclusions

*In situ* investigation of the structural evolution of polystyrene colloidal crystals during heating was carried out using a high-resolution small-angle X-ray scattering setup at the P10 beamline of PETRA III. The first experiments have provided new insight, allowing for better understanding of the phenomena that take place during the thermal degradation of colloidal crystals. In particular, at the first stage of heating a rearrangement of crystalline domains was observed, which is apparently related to a heat-driven redistribution of strains in thin films of colloidal crystals. Further temperature rise resulted in thermal shrinkage of the colloidal crystal lattice. The transition from crystalline to amorphous phase occurs in a narrow temperature interval for both investigated colloidal films and is accompanied by the transformation of the particle shape from a sphere to a dodecahedron. The melting transition of the colloidal crystal consisting of larger particles of 430 nm size took place at higher temperature than the transition temperature of the crystal made of smaller particles of 163 nm size. This is in agreement with previous studies on size- and free-surface-dependent behaviour of the glass transition temperature in polymer films (Keddie *et al.*, 1994 ▶; Bäumchen *et al.*, 2012 ▶). To reveal more details of the melting transition in colloidal crystals of various thicknesses, particle sizes and growth parameters further experiments exploring coherent X-ray diffraction imaging are planned.
